# Towards a Sustainable Public Transportation: Replacing the Conventional Taxis by a Hybrid Taxi Fleet in the West Bank, Palestine

**DOI:** 10.3390/ijerph17238940

**Published:** 2020-12-01

**Authors:** Fady M. A. Hassouna, Mahmoud Assad

**Affiliations:** 1 Civil Engineering Department, An-Najah National University, P.O. Box 7, Nablus 44830, Palestine; 2 Mechanical Engineering Department, An-Najah National University, P.O. Box 7, Nablus 44830, Palestine; m_assad@najah.edu

**Keywords:** sustainable public transportation, transportation in Palestine, environmental impact assessment, GHG emissions, greenhouse gas, hybrid vehicles

## Abstract

Recently, developing sustainable public transportation systems has been highlighted by decision makers and transportation agencies, due to the development of urban areas and the related environmental problems. Implementing new vehicle technologies has been introduced as an appropriate alternative to the conventional taxis. Hybrid electrical vehicles (HEVs) have been the potential candidates for replacing the conventional taxis, since they are more eco-friendly than conventional ones and even more reliable than electric vehicles (EVs) as a mode of public transportation. In this study, current and future environmental impact assessments have been determined for the taxi fleet in the West Bank, Palestine, and the implications of using new vehicle technologies (hybrid taxis) as a replacement of the conventional taxi fleet have been investigated. In order to perform this study, firstly, the data of the number of taxis for the period of 1994–2018 have been collected and a prediction model for the future number of taxis has been developed. The expected total amounts of consumed fuels have been then estimated. Finally, the current and the future N_2_O and CO_2_, and emissions, have been estimated and the expected influences of hybrid taxis have been determined. The results of the analysis have concluded that replacing 50% of conventional taxis with a hybrid fleet could achieve 42.3% and 28% reductions in N_2_O and CO_2_, respectively, in the next 10 years. A 395% increase in CH_4_ could be obtained due to the higher amount of CH_4_ that is produced by the gasoline combustion compared to the diesel fuel, since hybrid vehicles have gasoline-based engines (GHG in terms of CO_2_-equivalent could be increased by 28.2%).

## 1. Introduction

During the last two decades, the transportation sector has played a key role in developing trading, industry, and societies in general. The fast development of the urban areas has led to an increasing demand on public transportation. More specifically, in regions with no subway networks or weak bus lines, the highest demand has been observed for taxis.

On a worldwide scale, 26% of the total consumed energy is used by the transportation sector and 23% of GHG emissions are energy-related [1,2]. The public transportation sector is one of the leading contributors to environmental issues, due to the high number of daily kilometers driven and to the long period of working time for the modes of transportation, especially taxis.

Due to the strict gas emissions laws in the big cities and the high number of travelled-kilometers by taxis, the need to improve the taxi fleets using new eco-friendly technologies has become a very urgent issue [3]. Hybrid electric vehicles (HEVs), plug-in hybrid electric vehicles (PHEVs), and full electric vehicles (EV) are technologies that could provide solutions to reduce the dependency on fossil fuel-based energy and to decrease GHG emissions [4].

The hybrid vehicle is the potential candidate among these new technologies, due to the developed powertrain, less depletion of the battery, and the longer battery-lifetime compared to full EVs [4]. Moreover, in some countries like Palestine, due to the high percentage of fossil fuel-based electric energy, which is up to 63% of the total electricity consumption [5], there would be no significant environmental benefits from using EVs. Furthermore, other factors such as topography, weather conditions, and charging time would affect the reliability of EVs in Palestine. Therefore, HEVs could be the most potential alternative to conventional vehicles in Palestine.

Generally, four driving modes are available in HEVs in order to reduce fuel consumption and gas emissions. (1) Electric mode is the default mode of the vehicle that is activated by default in order to allow the driver to operate the vehicle electrically when the status of the electric system allows it, and this happens when the battery is sufficiently charged. (2) Hybrid mode is the mode that allows the vehicle to be operated electrically at low and medium speeds based on the charge level of the battery, while the internal combustion engine (ICE) is switched off in this instance, but it will be switched on during the fast acceleration or when the battery charge level is too low. (3) Hold mode is the mode that allows the vehicles to be operated by the ICE, and the electric motors to supply the 12-volt electrical system without charging the battery. (4) Charging mode allows the vehicle to be operated by the ICE, the electric motor to supply the 12-volt electrical system, and to charge the battery fully [5].

In Palestine, due to the weakness of bus lines, the absence of subway networks, and other cultural issues, Palestinians mainly depend on taxis rather than any other public transportation modes. Therefore, taxis play a key role in the public transportation sector. In order to develop a sustainable public transportation system, conventional taxi fleets should be replaced by other vehicle technologies. HEV will be the most appropriate alternative, due to the aforementioned reasons.

In this study, the environmental impact assessment of taxis in the West Bank, Palestine for the years 2020 and 2030 were determined, and the implications of future replacement of conventional taxis by hybrid vehicles have been determined. Since that HEV is still a very new technology in the Palestinian market, the future of this technology is unexpected, especially due to the absence of governmental strategies. Therefore, the future implications of the hybrid taxis during the next 10 years (in 2030) have been estimated based on two scenarios. (1) In case there is no clear plan, it could be implemented by the government in order to replace the conventional taxi fleets; the expected penetration rate of the hybrid taxis has been assumed to be 10% (case 1) during the next 10 years (in 2030), which is the normal increase rate that could be obtained in the absence of any other external factors. (2) In case there is a clear plan that could be implemented by the government in order to replace the conventional taxi fleets, the expected penetration rate of the hybrid taxis has been assumed to be 50% (case 2) during the next 10 years (2030), which is the maximum expected value that could be obtained due to the unstable economic and political situations in Palestine that form an obstacle in front of the governmental strategies. Hence, these two values (10% and 50%) present the minimum and maximum expected values or boundaries.

In order to conduct the study, a prediction model for the number of taxis has been developed based on the total number of taxis for the period of 1994–2018. Next, the total travelled kilometers and total consumed fuels by taxis in 2020 and 2030 have been determined. Finally, total GHG emissions and environmental implications of hybrid taxis have been determined. The results have showed the significant environmental benefits that could be gained by replacing conventional taxi fleets by hybrid ones.

Since the percentage of the hybrid vehicles in Palestine is still less than 1% (officially neglected) [5] and no studies have assessed the future environmental impacts of the public transportation sector, there is a need to have a current and a future environmental impact assessment as a first step for any future strategies or plans related to public transportation sector. This study will pave the way for further comprehensive studies that would target the public transportation sector in Palestine, and more specifically in the West Bank, which is the largest province (based on area) that includes 16 cities with a total population of 3.05 million [6].

## 2. Literature Review

Several studies have globally addressed the environmental impacts of public transportation sector and the expected influences of using new vehicle technologies as a replacement to conventional modes of public transportation. However, in Palestine, there are still not enough studies and information about the expected implications of using the new vehicle technologies in the public transportation system, since these new technologies, such as HEVs, have entered the market recently due to the restrictions on vehicle exporting during the last 10 years. Therefore, there is still not enough information about hybrid vehicles’ implications and the percentage of hybrid vehicles is still less than 1% (neglected by officially announced data).

One of the studies that has addressed this issue has been conducted in Iran by Ahmadabadi et al. [7]. In this study, the environmental impacts of replacing conventional taxis with a hybrid fleet in Tehran, Iran have been assessed. In order to perform this study, data related to air quality, pollution, and taxi distribution in the city have been collected and different design vehicles have been used to evaluate the emissions and the technical performance. The study has recommended that a conventional fleet of taxis should be replaced by HEVs. In addition, the results have showed that the Toyota Prius could be the best alternative for replacing the current conventional taxis in terms of cost and emissions.

In Portugal, a study has been conducted by D’orey et al. [8] in order to evaluate the usage of taxi-sharing systems to reduce the environmental impacts of taxi operation in Porto, Portugal. This study has presented a realistic and a large-scale evaluation of the suggested system. This model has been sub-divided into three parts (taxi-sharing algorithm, operation routing, and system state). The suggested approach has improved the efficiency of taxis by determining the best match between taxi user requests, which has led to a reduction in the total and vacant travel distance. Moreover, the results have showed that, with full deployment of this system, a 9% reduction in CO_2_ has been expected.

In the United Kingdom, the replacement of London city taxis with a hydrogen fuel cell hybrid has been evaluated by Baptista et al. [4]. The study has introduced a life cycle analysis for taxis in terms of energy consumption and CO_2_ emissions, focusing on the impacts of alternative vehicle technologies for taxis. The study has compared the CO_2_ emissions and the energy consumption of hydrogen fuel cell hybrid taxis with the ICE diesel taxis. The results have showed that the fuel cell powered-vehicles have lower energy consumption (4.34 MJ/km) and CO_2_ emissions (235 g/km) than Internal combustion engine vehicles (ICEVs) (9.54 MJ/km and 738 g/km).

In Bulgaria, a study has been conducted by Grozev et al. [9]. It has aimed to estimate the expected amount of CO_2_ emissions by the taxi fleet of Ruse, Bulgaria. In this study, CO_2_ emissions of conventional 25 taxicabs have been evaluated, and then the results have been compared to the ones that have used newer technologies. The study has recommended that EV taxis could be a better alternative to a conventional fleet taxi in Ruse case, based on several factors.

Another study has been conducted in Portugal by Castel-Branco et al. [3]. It has presented a suggested method to find the most efficient hybrid powertrain that could produce less emissions and less energy consumption as an alternative to a conventional taxi fleet in Lisbon, Portugal. It has been concluded that hybrid powertrains have revealed to be more advantageous compared to conventional taxis. More specifically, using hybrid vehicles could reduce energy consumption by up to 47% in urban conditions, and could reduce life cycle gas emissions by 49%.

Likewise, a study has been conducted in Russia by Kapustin and Rakov [10] in order to evaluate the environmental and economic efficiency of vehicles with different types of engines. The environmental impacts have been assessed at all the stages of the life cycle. The results have concluded that the most significant reductions in CO and NOx emissions have been obtained when hybrid technologies have been used. Moreover, the operation costs have been reduced by 28% when hybrid vehicles have been used.

Similarly, a study has been conducted by Nordelof et al. [11] to determine the environmental impacts of HEV compared to other types of vehicles. The study was conducted based on a trade-off between the benefits when operating the vehicles and possible new or increased negative impacts from production and from energy supply. The relevant impacts were determined using life cycle assessment. The results of study indicated that hybridized powertrains are beneficial in congested traffic where the many stops allow for regenerative breaking to recover energy.

In China, a study has been conducted by Ha [12] in order to compare the environmental impacts of conventional vehicles with HEVs and EVs. A life cycle assessment with GaBi6 software was established. The results of the study indicated that the global warming potential, abiotic depletion potential, and ozone depletion potential of HEVs are 78%, 65%, and 85% of the conventional vehicles, respectively.

Another study was conducted in Italy by Lombardi et al. [13] in order to compare the environmental impact assessment of EVs, plug-in hybrid gasoline-electric vehicles, plug-in hybrid fuel cell-battery vehicles, and conventional vehicles. In this study, the impacts from the powertrain production, vehicle use phase, and powertrain end of life were compared. The study compared four powertrain scenarios maintaining the same chassis. Next, a sensitivity analysis on different energy mixes has been included. The results concluded that, in the case of fuel depletion, climate change, and cumulative energy demand, the lowest value corresponds to the plug-in hybrid gasoline-electric vehicle, followed by the plug-in hybrid fuel cell-battery vehicle, the pure electric, and finally the conventional gasoline vehicle. Moreover, the source of the electricity significantly affects the benefits of using EVs and PHEVs.

The majority of the previous studies have assessed the environmental and energy implications of HEV compared to conventional and other new vehicle technologies such as EVs and hydrogen fuel vehicles. In Palestine, recently, HEVs have started to penetrate the market, while other vehicle technologies such as EVs, PHEV, and hydrogen fuel vehicles seem to be neglected in all future policies and strategies, due to the absence of charging infrastructures, unstable electric grid, and the absence of required technologies. Therefore, in this study, environmental impact assessments of hybrid taxis were determined compared to conventional taxis. This study would offer the basic information required for decision makers in order to develop clear and efficient strategies towards a sustainable public transportation sector and to pave the way for further comprehensive studies. Therefore, the current and the future environmental impacts of the taxi fleet in West Bank, which is the largest province in Palestine based on area, have been assessed and the implications of using HEV as a replacement for conventional taxis have been determined.

## 3. Data and Methods

In this study, the required were mainly collected from the Palestine Central Bureau of Statistics. Data related to number of taxis for the period of 1994–2018 were obtained from the Palestinian Central Bureau of Statistics (2020) [6]. The total annual travelled kilometers by gasoline and diesel taxis have been obtained from the Palestinian Central Bureau of statistics [14]. The fuel efficiency rates (l/km) of taxis have been obtained from the Palestinian Central Bureau of Statistics [15].

In 1993, as a result of the Oslo peace accord between Palestinian Liberation Organization and Israel, Palestinian National Authority (PNA) was created. Based on this agreement, from 1994, the PNA has been in charge of controlling the West Bank and Gaza Strip, Palestine [16,17]. Therefore, before this date (1994), the data had been recorded based on different criteria and, for this reason, the period of 1994–2018 has been selected.

**Figure 1:**
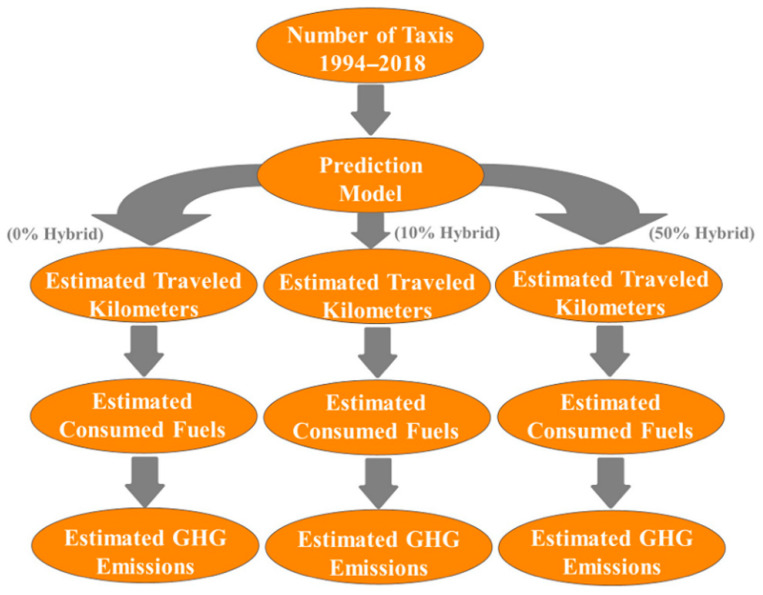
Implemented methodology process.

Firstly, a prediction model for the number of taxis has been developed based on the total number of taxis for the period of 1994–2018. Secondly, the total fuel consumption has been determined based on the total traveled kilometers. Thirdly, total GHG emissions produced by taxis in 2020 (0% hybrid taxis penetration) and 2030 based on two scenarios (10% and 50% hybrid taxis penetration) have been estimated, as shown in the implemented process in Figure 1.

### 3.1. Number of Taxis Prediction Model

In the first stage, a prediction model for the number of taxis has been developed based on the total number of taxis for the period of 1994–2018, using Holt’s Exponential Smoothing modeling method, which is the most appropriate modeling method for this case based on the analysis of IBM SPSS 25 software. In addition, other prediction models, such as ARIMA model, require a large sample size up to 50 years in order to achieve a high accuracy prediction [18]. However, the number of taxis in Palestine is controlled by the ministry of transportation who registers the taxis (by selling permits); this number still depends on the demand for taxis; and the future number of taxis can be predicted based on the differences in the number of taxis during the previous years.

Exponential Smoothing is a technique of time series in which data over time is smoothed exponentially either by assigning exponentially increasing or decreasing weights with the data points. There are three methods of Exponential Smoothing: Single Exponential Smoothing, Double or Holt’s Exponential Smoothing, and Triple or Winters Exponential Smoothing [19].

Smoothing the time series is the main principle of the exponential smoothing methodology. Next, the smoothed time series is used in prediction the future values. In this method, the recent data in the time series have a greater effect on the predicted values and farther back data have less effects on the prediction process. The equation of Holt’s exponential smoothing can be presented as in Equation (1) [20]:

F_t+m_ = s_t_ + mb_t_ (1)

s_t_ = αx_t_ + (1 − α)(s_t−1_ + b_t−1_) (2)

b_t_ = β(s_t_ − s_t−1_) + (1 − β)b_t−1_ (3)

where α is the smoothing factor ( 0 < α< 1), and β is the trend factor, (0 < β < 1), F is an estimating of x value at time t + m, b_t_ is the best estimation of the trend at time specific year t, x_t_ is the sequence of data, s_t_ is the smoothed value for year t, and m is a value greater than 0.

### 3.2. Fuel Consumption Estimationn

In the second stage, total traveled kilometers by taxis in 2020 and 2030 have been determined based on the predicted number of taxis in 2020 and 2030 (using the predation model) and the total traveled kilometers by taxis in 2014, which is the only available yearly data. Next, depending on the total traveled kilometers by taxis in 2020 and 2030 and using the average fuel consumption rate of taxis in Palestine (L/km), the expected total amounts of fuel consumption by taxis in 2020 (0% hybrid taxis) and 2030 (based on two scenarios: 10% hybrid and 50% hybrid taxis) have been estimated.

### 3.3. GHG Emissions Estimation

In the third stage, the total expected amounts of CH_4_, CO_2_, and N_2_O emissions in 2020 have been determined based on the total estimated consumed diesel fuels by taxis in 2020 and using the average amounts of emissions produced per liter of diesel due to the combustion in ICEs (kg of emission per liter of diesel). Similarly, the total expected amounts of CH_4_, CO_2_, and N_2_O emissions in 2030 have been determined based on the total estimated consumed diesel and gasoline fuels by taxis in 2030 (based on two scenarios) and using the average amounts of emissions produced per liter of diesel and gasoline due to the combustion in ICEs (kg of emission per liter of diesel and gasoline). Finally, the implications of partial replacement of conventional taxis by hybrid fleets have been determined by comparing the first scenario (10% hybrid penetration) and second scenario (50% hybrid penetration) with the benchmark scenario (0% hybrid penetration).

## 4. Data Analysis and Discussion

To determine the GHG emissions produced by taxis in the West Bank, and to determine the implications of replacing a conventional taxi fleet with a hybrid one, a prediction model for the number of taxis has been developed. Next, the total travelled kilometers, the total amount of fuel consumption, and the total GHG emissions in 2020 and 2030 were estimated based on two scenarios.

### 4.1. Number of Taxis Prediction Model

In the first stage, a prediction model has been developed. It has been used to estimate the total number of taxis in 2020 and 2030. The prediction model has been developed using the Holt’s Exponential Smoothing Method and using data related to number of taxis for the period of 1994–2018.

Based on the number of vehicles time series for the subject period, there has almost been an increasing trend in the number of registered taxis in the West Bank during 1994–2018 except for the period of 2000–2003, due to the second Palestinian uprising against Israel, which began in 2000. In this period, many taxi vehicles could not be officially recorded due to the absence of the annual registration process in some cities.

In addition, the results have showed that there was a slight decrease in the number of registered taxis during the period 2014–2018 due to the economic issues that made some taxis’ owners unable to renew the annual registration (permits) of their vehicles.

Using IBM SPSS 25 software, the best fit Holt’s Exponential Smoothing model has been selected based on the minimum Mean Absolute Error (MAE) value. The statistics and the parameters for the selected model are shown in Table 1.

**Figure 2:**
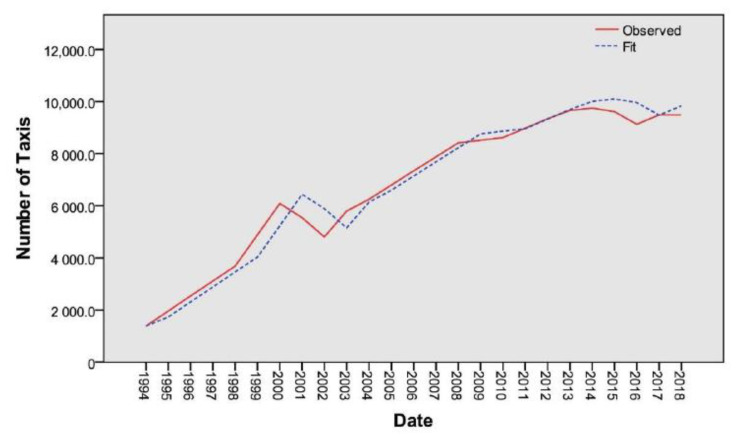
Prediction model for the number of taxis in the West Bank (observed and fit).

The developed prediction model statistics has showed that the prediction accuracy of the model is acceptable and can be used for predicting the number of taxis with no reservation (the observed and fit-values of the developed model are shown in Figure 2). As a result of applying the developed model, the predicted total number of taxis in 2020 and 2030 are 10,182 and 13,654, respectively. Although there is an obvious time lag in the prediction of the number of taxis, the model was developed based on the yearly data for 25 years, which is sufficient to predict the next 10 years of data with acceptable accuracy as recommended by several previous studies, including Hassouna and Al-Sahili [5] and Singh et al. [19]. Moreover, in the exponential smoothing prediction method, the recent data in the time series have a greater effect on the predicted values, and farther back data have less effects on the prediction process, which means that the accuracy of the prediction depends mainly on the recent data.

### 4.2. Fuel Consumption Estimationn

In the second stage, based on the number of taxis in 2014, which was 9749, and the predicted number of taxis in 2020 (10,182), there is a 4.4% increase in the number of taxis in 2020 compared to 2014. Therefore, using the same percentage of increase and based on the total travelled kilometers in 2014, which was 490 million km [14], the total expected travelled kilometers in 2020 by taxis could be estimated (511.56 million km). Similarly, based on the number of taxis in 2014, the predicted number of taxis in 2030 (13,654), and using the same growth rate, the expected total travelled kilometers by taxis in 2030 can be estimated (657.1 million km).

In the third stage, based on the calculated number of travelled kilometers by taxis in 2020 (511.56 million km) and 2030 (657.1 million km), and using the average fuel consumption rate of taxis in Palestine, which is 0.099 L/km [14], the estimated consumed amount of fuels by taxis in 2020 and 2030 could be estimated (almost all taxis in Palestine are diesel ICEVs with neglected percentage of gasoline and hybrid vehicles). Thus, the estimated total amount of diesel that could be consumed by taxis in 2020 is 50.65 million liters.

In 2030, due to the continuous improvement in fuel efficiency of the manufactured engines during the next 10 years (assuming that average fuel consumption in 2030 will be decreased by 15% compared to 2020), the expected average diesel efficiency rate for taxis is 0.089 L/km. Likewise, the average gasoline consumption rate for the hybrid vehicles (all the produced HEVs are gasoline-based hybrid) is 0.055 L/km based on the Fuel consumption Report [21]. Assuming that the average gasoline consumption rate for hybrid vehicles in 2030 will be decreased by 20% due to the continuous improvement in fuel efficiency of HEVs in the next ten years, the expected average gasoline consumption rate could be 0.044 L/km in 2030. The reduction values in fuel consumption (15% for conventional vehicles and 20% for HEV) were selected based on a study by Lang et al. [22], in China, which concluded that the estimated average fuel consumption rate for conventional and HEVs would be reduced by 15% and 20%, respectively, during a period of 10 years due to the continuous improvement in engine manufacturing. Moreover, the same reduction values (15% for conventional vehicles and 20% for HEV) were selected by Hassouna and Al-Sahili [16] as the most appropriate values for vehicles in Palestine during the next 10 years. Therefore, using the calculated total travelled kilometers by taxis in 2030 (657.1 million km), the mentioned expected average fuel efficiency rate (L/km) for taxis in 2030, and, based on the two scenarios, the expected total fuel consumption could be estimated, as shown in Table 2.

### 4.3. GHG Emissions Estimation

Finally, based on the estimated total consumed fuels by taxis and using the average rates of gas emissions produced by diesel (conventional taxis) and gasoline (hybrid taxis) combustion by the ICEs, current and future (2030) GHG emissions have been estimated. Moreover, the implications of partial replacement of conventional taxis by hybrid fleet have been determined.

The average amounts of CH_4_, N_2_O, and CO_2_ emissions produced by the gasoline ICEs are 0.72, 0.05, and 2597.87 gm/L, respectively [5]. The average amounts of emissions produced by diesel ICEs are 0.04, 0.16, and 2924.90 gm/L, respectively [5]. Therefore, based on the expected total amount of consumed diesel in 2020, the expected CH_4_, N_2_O, and CO_2_ emissions in 2020 are 2.0, 8.1, and 148,146.2 tons, respectively, which are equal to 150,610.00 tons of CO_2_-equivalent of GHG (CO_2_-equivalent conversion factors: 1 kg CH_4_ = 25 kg CO_2_, 1 kg N_2_O = 298 kg CO_2_), as shown in Table 3.

Similarly, using the total consumed amounts of gasoline and diesel in 2030 (from Table 3), and based on scenario #1, the expected CH_4_, N_2_O, and CO_2_ emissions are 4.21, 8.565, and 161,445.34 tons, respectively, which are equal to 164,102.96 tons of CO_2_-equivalent of GHG. However, based on scenario #2, the expected CH_4_, N_2_O, and CO_2_ emissions are 11.58, 5.4, and 123,118.53 tons, respectively, which are equal to 125,017.23 tons of CO_2_-equivalent of GHG, as shown in Table 4.

The results for the two scenarios compared to the benchmark case are summarized in Table 5. It can be noticed that replacing 10% of conventional taxis with a hybrid fleet could achieve 5.6% and 8.5% drops in CO_2_ and N_2_O emissions, respectively. However, a 79.9% increase in CH_4_ could be obtained due to the higher amount of CH_4_ produced by gasoline combustion, which is used by the hybrid ICEs compared to the diesel fuel (the total GHG in terms of CO_2_-equivalent could be decreased by 5.65%). Whereas replacing 50% of conventional taxis with the hybrid fleet could achieve 28% and 42.3% reductions in CO_2_ and N_2_O, respectively, while a 395% increase in CH_4_ could be obtained (the total GHG in terms of CO_2_-equivalent could be decreased by 28.2%). Moreover, the estimated total GHG emissions in terms of tons of CO_2_-equivalent in 2020 is 150,610, while the total GHG emissions in terms of tons of CO_2_-equivalent in 2017 (the last officially announced data) by energy sector was 3,392,400 [6], which is equal to 4.44% of the energy sector in 2017.

## 5. Conclusions

In this study, the current and the future (2030) environmental impact assessment of taxi fleets in the West Bank, Palestine, have been determined and the implications of the partial replacement of conventional taxis by the hybrid fleets has been investigated. The GHG emissions were determined based on the expected total fuel consumption by taxis that have been predicted using the exponential smoothing model. Due to the absence of studies that address the sustainability of public transportation in Palestine, this study could pave the way for further future comprehensive studies. Based on the results of this study, the following findings are offered:

- The estimated total GHG emissions produced by taxis in the West Bank, in 2020, is 150,610 Tons (in term of CO_2_ equivalent). This value constitutes more than 4.4% of the total GHG emissions produced by the energy sector, which is relatively a high percentage compared to the small number of taxis in the West Bank.
- The expected rate of diesel fuel consumption and GHG emissions by the taxi fleet in 2020 is relatively high compared to those in developed countries due to the high percentage of old taxis that are still in service. The expected total diesel fuel consumption is 50.65 million liters. However, the expected CH_4_, N_2_O, and CO_2_ emissions are 2.34, 9.36, and 171,048.2 tons, respectively.
- In 2030, in case the conventional taxis are still used without any replacement by a new vehicle technology (such as hybrid vehicles), the expected diesel fuel consumption is 58.48 million liters. However, the expected CH_4_, N_2_O, and CO_2_ emissions are 2.34, 9.36, and 171,048.2 tons, respectively, which means a significant increase in total GHG in terms of CO_2_-equivalent by 15.5% compared to 2020.
- In 2030, based on the first scenario (10% penetration of the hybrid fleet), the expected CH_4_, N_2_O, and CO_2_ emissions are 4.21, 8.565, and 161,445.34 tons, respectively, which are equal to 164,102.96 tons of CO_2_-equivalent of GHG. This means a 5.65% reduction in total CO_2_-equivalent of GHG compared to the 0% hybrid taxis scenario.
- Replacing 50% of the conventional taxis with the hybrid fleet could achieve 28% and 42.3% reductions in CO_2_ and N_2_O, respectively, in 2030, while a 395% increase in CH_4_ could be obtained due to the higher amount of CH_4_ produced by gasoline combustion compared to diesel fuel (the total GHG in terms of CO_2_-equivalent could be decreased by 28.2%).
- It is recommended to include buses in any future environmental impact assessment study. In addition, CO and SO_2_ gases emissions should be investigated and measured, since these two items could not be included in this study due to the unavailability of data.
- This study has assumed that the fuel efficiency of the hybrid vehicles would be increased by 20% (20% reduction in fuel consumption) during the next 10 years. Therefore, in case an unexpected new technology is used to improve the fuel efficiency, the results of this study should be recalculated in order to be valid. Moreover, the amounts of gas emissions have been determined based on the current generation of engines and, therefore, in case a new generation of engines is developed during the next 10 years, the GHG emissions should be recalculated.

## Figures and Tables

**Table 1:** Prediction model statistics.

Model	Model Fit Statistics			Ljung–Box Q(18)		
	R-Squared	MAPE	MAE	Statistics	DF	Sig
Holt’s Model	0.968	6.171	344.0	11.663	16	0.767
Prediction Model Parameters						
	Estimate	SE	t	Sig		
Alpha	1.0	0.215	4.641	0.0		
Beta	0.001	0.101	0.006	0.995		

**Table 2:** Expected total consumed fuels by taxis in 2030.

Scenarios	Diesel (Million Liters)	Gasoline (Million Liters)
Scenario #0 (100%ICEVs + 0% Hybrid)	58.48	0
Scenario #1 (90% ICEVs + 10% Hybrid)	52.63	2.89
Scenario #2 (50% ICEVs + 50% Hybrid)	29.25	14.46

**Table 3:** Estimated GHG emissions by taxis in 2020.

Emissions	Diesel ICE Taxis	GHG (CO_2_-Equivalent in Tons)
CH_4_ (ton)	2.0	150,610.00
N_2_O (ton)	8.1	
CO_2_ (ton)	148,146.2	

**Table 4:** Estimated GHG emissions by taxis in 2030.

Scenarios	GHG Emissions	Diesel (ICE Taxis)	Gasoline (Hybrid Taxis)	Total	Total GHG (Tons of CO_2_-Equivalent)
Scenario 0 (100%ICE Taxis + 0% Hybrid)	CH_4_ (ton)	2.34	0	2.34	173,895.98
	N_2_O (ton)	9.36	0	9.36	
	CO_2_ (ton)	171,048.2	0	171,048.2	
Scenario 1 (90% ICE Taxis + 10% Hybrid)	CH_4_ (ton)	2.11	2.10	4.21	164,102.96
	N_2_O (ton)	8.42	0.145	8.565	
	CO_2_ (ton)	153,937.5	7507.84	161,445.34	
Scenario 2 (50% ICE Taxis + 50% Hybrid)	CH_4_ (ton)	1.17	10.41	11.58	125,017.23
	N_2_O (ton)	4.68	0.72	5.4	
	CO_2_ (ton)	85,553.33	37,565.2	123,118.53	

**Table 5:** Results summary for different scenarios compared to scenario 0 (0% Hybrid), 2030.

Scenarios	GHG Emissions	Total (%)	Total GHG (Tons of CO_2_-Equivalent) (%)
Scenario 1 (10% Hybrid Taxis)	CH_4_ (ton)	+79.9	−5.65
	N_2_O (ton)	−8.5	
	CO_2_ (ton)	−5.6	
Scenario 2 (50% Hybrid Taxis)	CH_4_ (ton)	+395	−28.2
	N_2_O (ton)	−42.3	
	CO_2_ (ton)	−28.0	
